# Supplementary material for: Jag1-Notch cis-interaction determines cell fate segregation in pancreatic development
Source: Nat Commun. 2023 Jan 21;14:348. doi: 10.1038/s41467-023-35963-w (PMC9867774; doi:10.1038/s41467-023-35963-w)
Supplement: Supplementary file 1 — Supplementary Information [file 41467_2023_35963_MOESM1_ESM.pdf]

Supplemental Information

Jag1-Notch *cis*-interaction determines cell fate segregation in pancreatic development

Xiaochan Xu[1], Philip Allan Seymour[2,3], Kim Sneppen[1], Ala Trusina[1], Anuska la Rosa Egeskov-Madsen[2,3], Mette Christine Jørgensen[2,3], Mogens H. Jensen[1,*], and Palle Serup[2,3,*]

December 16, 2022

[1]The Niels Bohr Institute, University of Copenhagen, DK-2100 Copenhagen Ø, Denmark

[2]Novo Nordisk Foundation Center for Stem Cell Biology (DanStem), University of Copenhagen, DK-2200 Copenhagen N , Denmark

[3]Novo Nordisk Foundation Center for Stem Cell Medicine (reNEW), University of Copenhagen, DK-2200 Copenhagen N , Denmark

[*]Correspondence: palle.serup@sund.ku.dk (P.S.), mhjensen@nbi.ku.dk (M.H.J)

Supplementary Table 1: Information of primary antibodies

Goat polyclonal anti-Pdx1 (IF 1:10000, WB 1:5000)	Beta Cell Biology Consortium (BCBC)	Cat#AB2027
Guinea pig polyclonal anti-Sox9 (IF 1:2000)	BCBC (Kind gift from Dr. Ole Madsen)	N/A
Rabbit polyclonal anti-Sox9 (IF 1:1000)	Merck Millipore	Cat#AB5535
Rabbit polyclonal anti-Ptf1a (IF 1:3000)	BCBC	Cat#AB2153
Guinea pig polyclonal anti-Ptf1a (IF 1:5000)	Kind gift from Dr. Jane E. Johnson	Hori et al. 2008, Genes Dev 22: 166-78.
Mouse monoclonal anti-Nkx6-1 (IF 1:500)	DSHB	Cat#F55A10
Rabbit polyclonal anti-Nkx6-1 (IF 1:2000)	BCBC	Cat#AB1069
Rabbit polyclonal anti-Ngn3 (IF 1:4000 for sections, 1:10000 for whole mount)	BCBC	Cat#AB2011
Guinea pig polyclonal anti-Glucagon (IF 1:4000 for sections, 1:10000 for whole mount)	Millipore (Merck)	Cat#4031-01F
Rabbit polyclonal anti-Glucagon (IF 1:2000)	Cell Signaling Technology	Cat#2760
Rat monoclonal anti-E-Cadherin (Cdh1) (IF 1:1000)	ThermoFisher	Cat#13-1900
Sheep polyclonal anti-Dll1 (IF 1:200)	R&D Systems	Cat#AF3970
Goat polyclonal anti-Jag1 (IF 1:200, WB 1:500)	Santa Cruz Biotechnology	Cat#sc-6011
Rabbit monoclonal anti-Jag1 (IF 1:50)	Cell Signaling Technology	Cat#2620
Rabbit monoclonal anti-Hes1 (IF 1:200)	Cell Signaling Technology	Cat#11988
Mouse monoclonal anti-pan-Actin (WB 1:4000)	ThermoFisher	Cat#MA5-11869

Supplementary Table 1. Primary Antibodies used in this study.

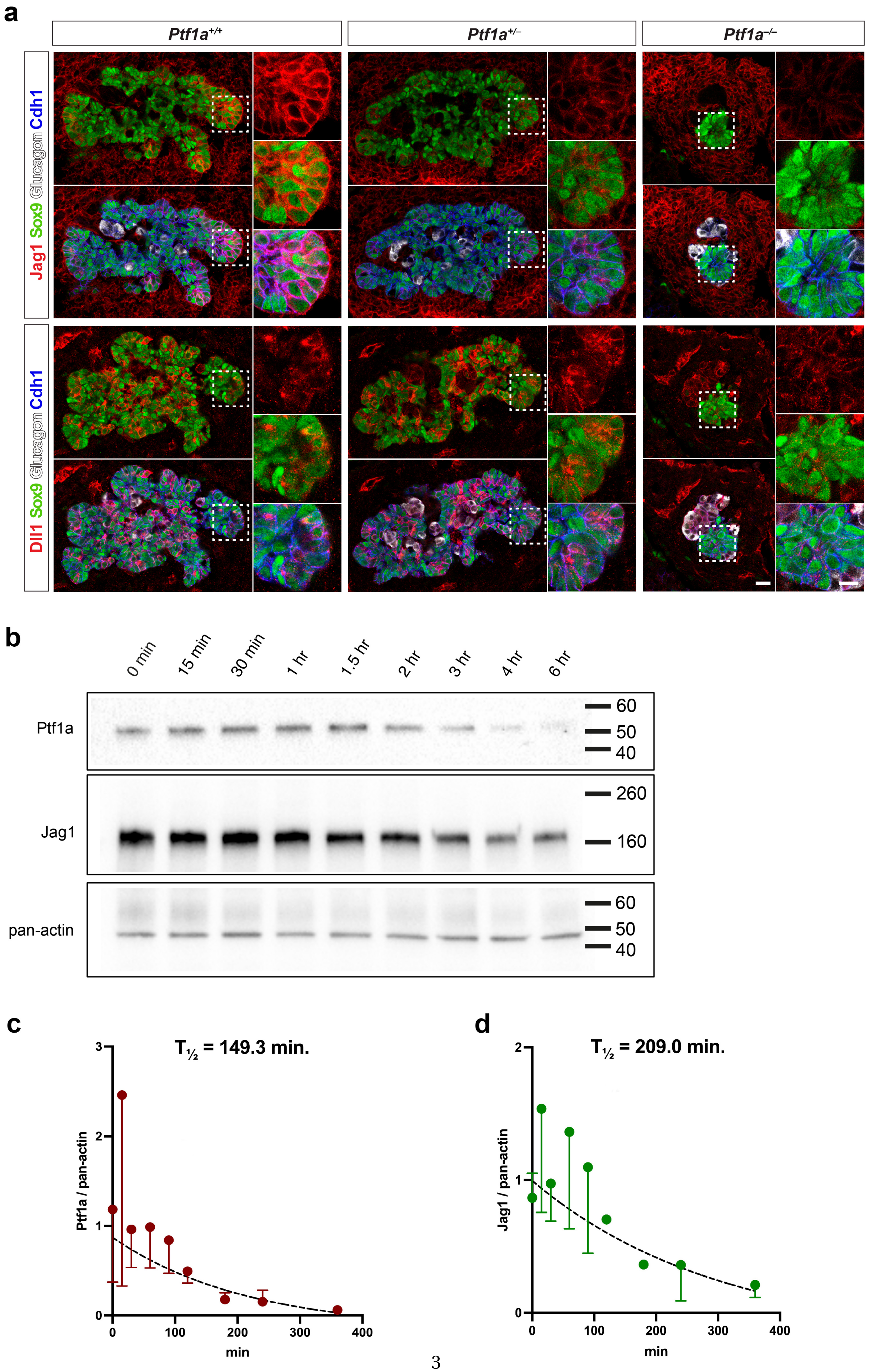
a
Ptf1a+/+
Ptf1a+/–
Ptf1a–/–
Jag1 Sox9 Glucagon Cdh1
Dll1 Sox9 Glucagon Cdh1
b
0 min
15 min
30 min
1 hr
1.5 hr
2 hr
3 hr
4 hr
6 hr
Ptf1a
Jag1
pan-actin
60
50
40
260
160
60
50
40
c
T½ = 149.3 min.
Ptf1a / pan-actin
min
0
100
200
300
400
d
T½ = 209.0 min.
Jag1 / pan-actin
min

Supplementary Fig. 1. Dependency of Jag1 and Dll1 on Ptf1a and Jag1/Ptf1a protein half-lives. a Expression of Sox9 (green), Cdh1 (blue), Jag1 and Dll1 ligands (red) in pancreas epithelium in E12.5 wildtype as well as heterozygous and homozygous *Ptf1a* mutant embryos. Scale bar: 20 μm. Insets show the boxed areas in higher magnification (also shown in Figure 1). Scale bar: 10 μm. The experiment was repeated with the same result on two sets of embryos. **b** Ptf1a and Jag1 protein stability in 266-6 cells. The cells were grown for 24 hours and then treated with 100 μM cycloheximide and samples were harvested at the indicated time points after addition of cycloheximide. Protein levels were analyzed by western blot with Actin serving as internal standard. **c** Relative Ptf1a levels normalized to Actin. Mean $\pm$ SD from three separate experiments. Dashed line: degradation curve fitted using robust regression. The calculated protein half-life is indicated. **d** Relative Jag1 levels normalized to Actin. Mean $\pm$ SD from three separate experiments. Dashed line: degradation curve fitted using robust regression. The calculated protein half-life is indicated.

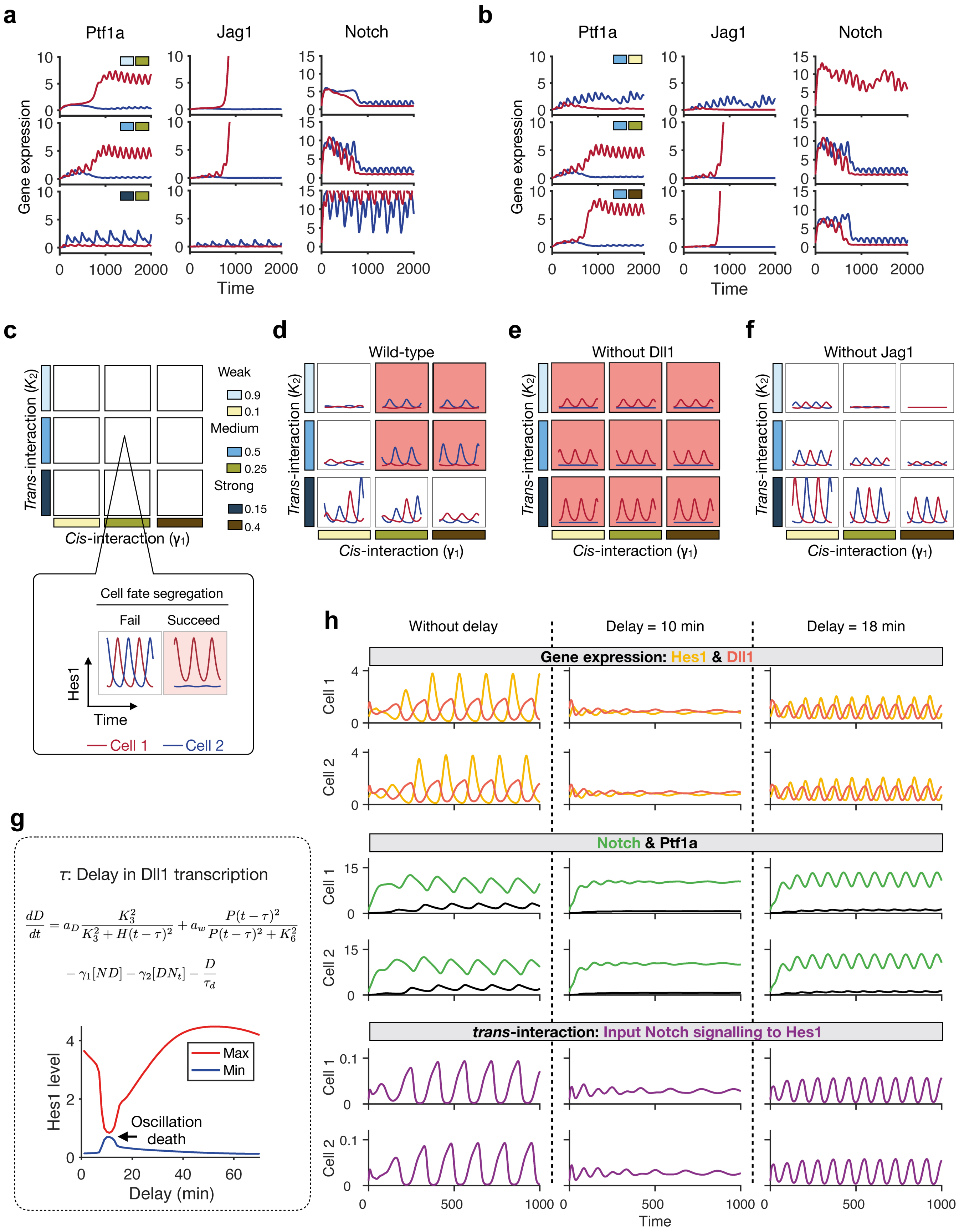
a
Ptf1a
Jag1
Notch
Gene expression
Time
b
Ptf1a
Jag1
Notch
Gene expression
Time
c
Trans-interaction (K2)
Cis-interaction (γ1)
Weak
0.9
0.1
Medium
0.5
0.25
Strong
0.15
0.4
Cell fate segregation
Fail
Succeed
Hes1
Time
Cell 1
Cell 2
d
Wild-type
Trans-interaction (K2)
Cis-interaction (γ1)
e
Without Dll1
Trans-interaction (K2)
Cis-interaction (γ1)
f
Without Jag1
Trans-interaction (K2)
Cis-interaction (γ1)
g
τ: Delay in Dll1 transcription
dD/dt = a_D K_3^2/(K_3^2 + H(t − τ)^2) + a_w P(t − τ)^2/(P(t − τ)^2 + K_6^2) − γ1[ND] − γ2[DN_t] − D/τ_d
Hes1 level
Max
Min
Oscillation death
Delay (min)
h
Without delay
Delay = 10 min
Delay = 18 min
Gene expression: Hes1 & Dll1
Cell 1
Cell 2
Notch & Ptf1a
Cell 1
Cell 2
trans-interaction: Input Notch signalling to Hes1
Cell 1
Cell 2
Time

Supplementary Fig. 2. Dll1 is important for MPC maintenance and Jag1 for cell fate segregation.

a–b Gene expression dynamics of Ptf1a, Jag1, and Notch in the two-cell models corresponding to weak, medium, and strong *trans*-interaction (**a**) or *cis*-interaction (**b**). The second rows in **a** and **b** are the same. **c** Schematic diagram for investigation of cell fate coexistence with different strengths of *trans*-interaction and *cis*-interaction. Values of K_2 and γ_1 used in the simulation of two interacting cells and dynamics of Hes1 expression in the late stage (time interval from 3,300 min to 3,600 min) are presented. **d** Final cell fates of two cells in wild type simulation with different strengths of *trans*- and *cis*- interaction. **e** Final cell fates of two cells when the expression of Dll1 is blocked by setting $a_D = 0$ and $a_w = 0$. **f** Final cell fates of two cells when the expression of Jag1 is blocked by setting $a_J = 0$. All of the subgraphs in **d**, **e**, and **f** are with the same coordinate axis. **g** Two-cell model with delay in Dll1 transcription (τ) recaptured "oscillation death". The delay caused a decrease in amplitude of Hes1 oscillation within a small time window of around 10 min without Jag1. **h** Dynamics of gene expression and activated notch signalling ($(\gamma_2[ND_t] + \gamma_2[NJ_t])^2/[(\gamma_2[ND_t] + \gamma_2[NJ_t])^2 + K_2^2]$) with different delays in Dll1 transcription. When the delay increases to 10 min, the oscillations are damped. When delay increases to 18 min, the oscillations appear again with a different frequency and also change from anti-phase to in-phase. Initial conditions for the two cells in all the two-cell models are the same: [0.5,1.1,0,1,0] and [0.6,1,0,1,0].

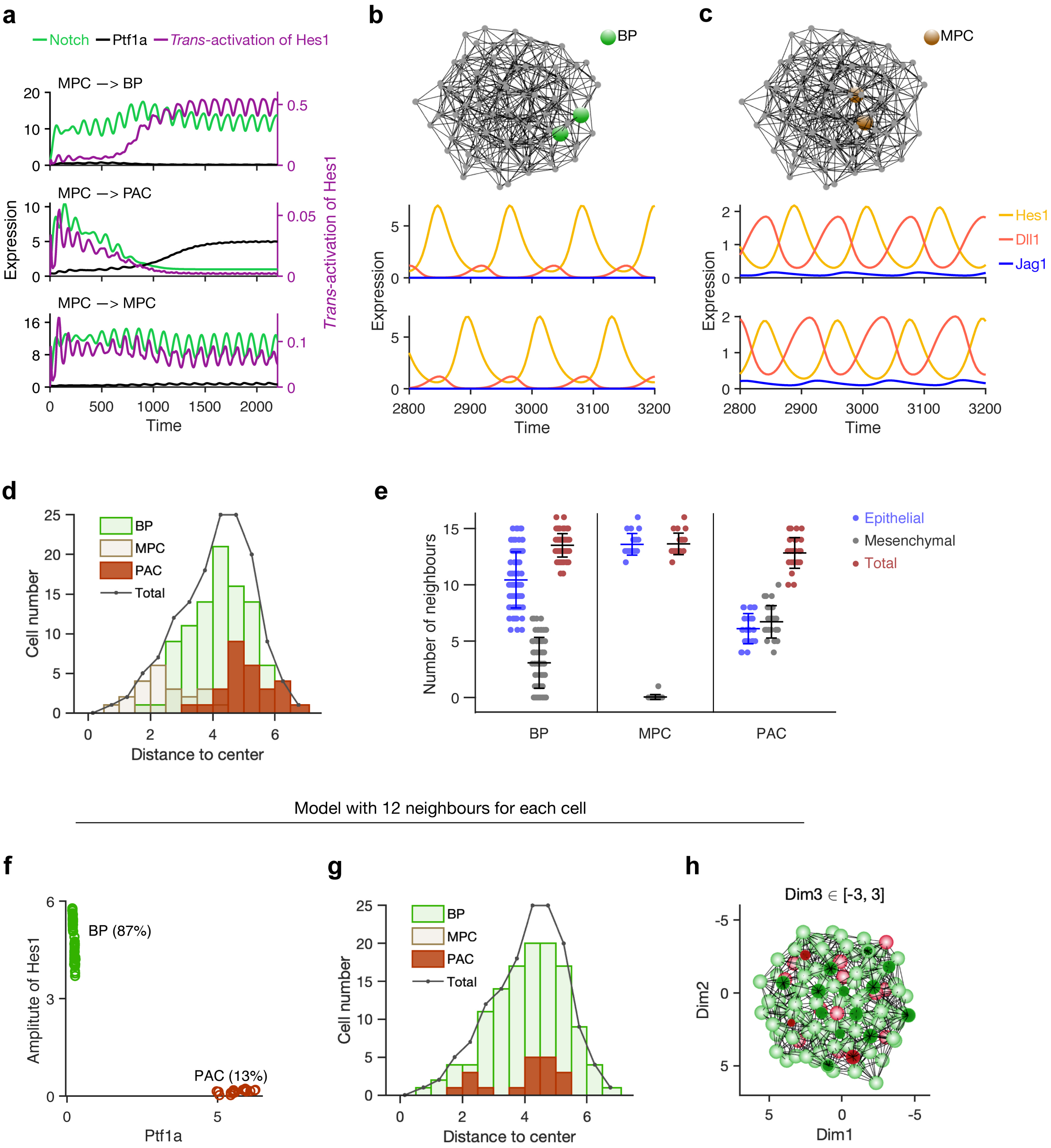
a
Notch
Ptf1a
Trans-activation of Hes1
MPC —> BP
MPC —> PAC
MPC —> MPC
Expression
Trans-activation of Hes1
Time
b
BP
Expression
Time
c
MPC
Hes1
Dll1
Jag1
Expression
Time
d
BP
MPC
PAC
Total
Cell number
Distance to center
e
Epithelial
Mesenchymal
Total
Number of neighbours
BP
MPC
PAC
Model with 12 neighbours for each cell
f
BP (87%)
PAC (13%)
Amplitute of Hes1
Ptf1a
g
BP
MPC
PAC
Total
Cell number
Distance to center
h
Dim3 ∈ [-3, 3]
Dim2
Dim1

Supplementary Fig. 3. Mathematical model for Notch signaling mediated MPC differentiation a Dynamics of expression of Ptf1a, Notch and activated notch signalling of cells shown in Figure 3b. **b–c** Positions and Expression dynamics of genes of interacting BP cells and MPC cells in the 3D structure are shown as examples of anti-phase oscillation of Hes1. **d** Statistics of cell numbers for each cell fate as the distance to the 3D structure center increases. **e** Statistics of numbers of neighbours for each cell fate. Mean $\pm$ SD, BP: N=93; MPC: N=22; PAC: N=28. **f–h** Simulation of the model with 12 epithelial neighbors for each cell. **f** Scatter plot of amplitude of Hes1 and Ptf1a expression of cells. **g** Cell fate distribution on 3D structure with a fixed number of neighbors. **h** Cross-section display of the 3D structure.

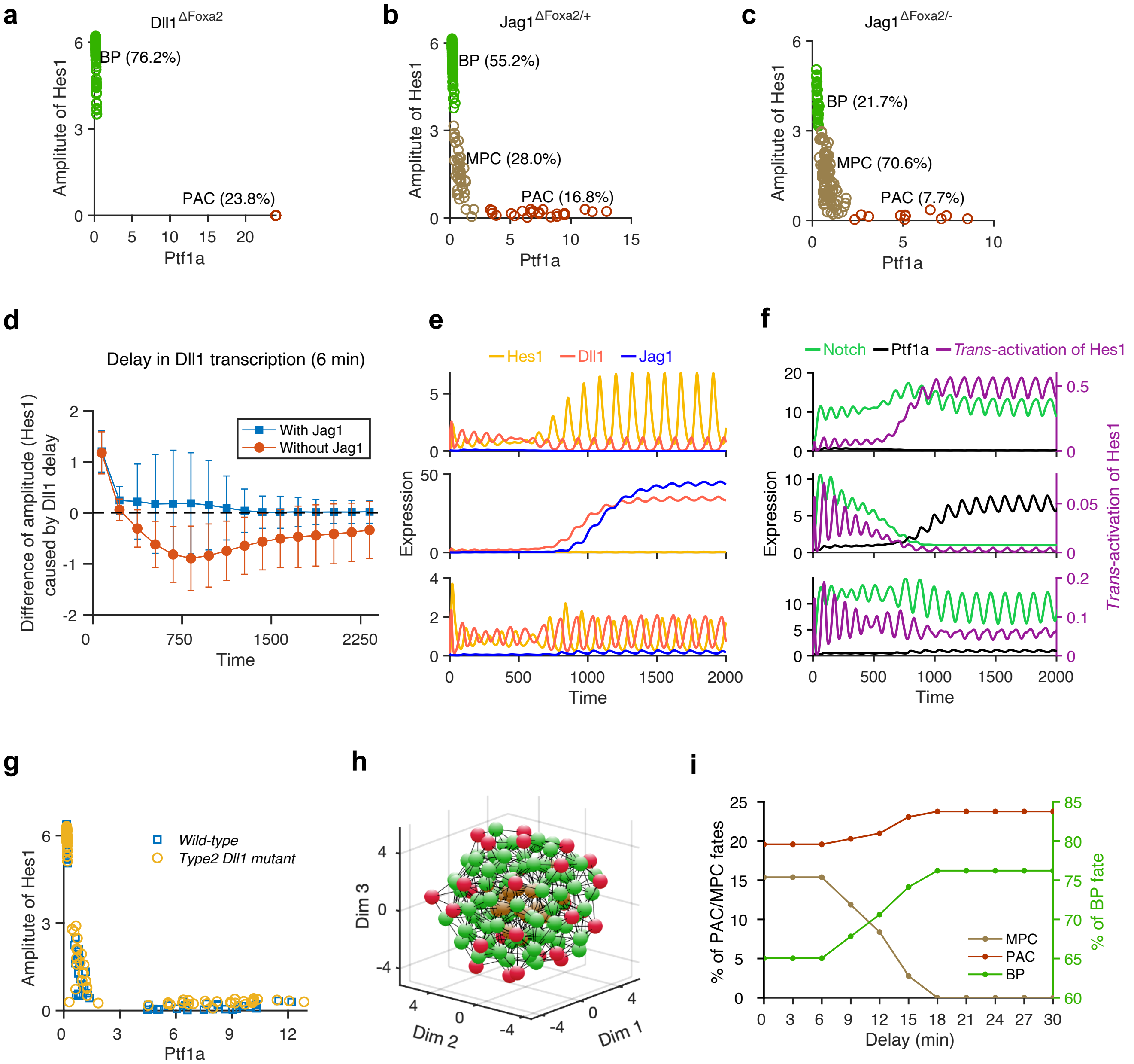

Supplementary Fig. 4. Delay in Dll1 transcription affects cell fate segregation. **a** Scatter plot of amplitude of Hes1 and Ptf1a level of cells in simulation with Dll1 deficiency. **b–c** Scatter plot of amplitude of Hes1 and Ptf1a level of cells in simulation with Jag1 deficiency. **d** In simulation, delay in Dll1 transcription does not reduce amplitude of Hes1 with Jag1 but reduce amplitude of Hes1 when Jag1 is blocked. Mean±SD, N=143. **e–f** Examples for dynamics of gene expression and notch signalling in *type2 Dll1* mutant simulation. **g** Scatter plot of amplitude of Hes1 and Ptf1a level of cells in *type2 Dll1* mutant simulation with 6 min delay in Dll1 transcription. **h** Cell fates distribution of *type2 Dll1* mutant simulation shown on 3D structure. **i** Cell fate proportions change as the delay in Dll1 transcription increases. The proportion of MPC fate decreases with the time delay while BP fate increases with the time delay. Simulations in **e–i** are done with Jag1 in the model.

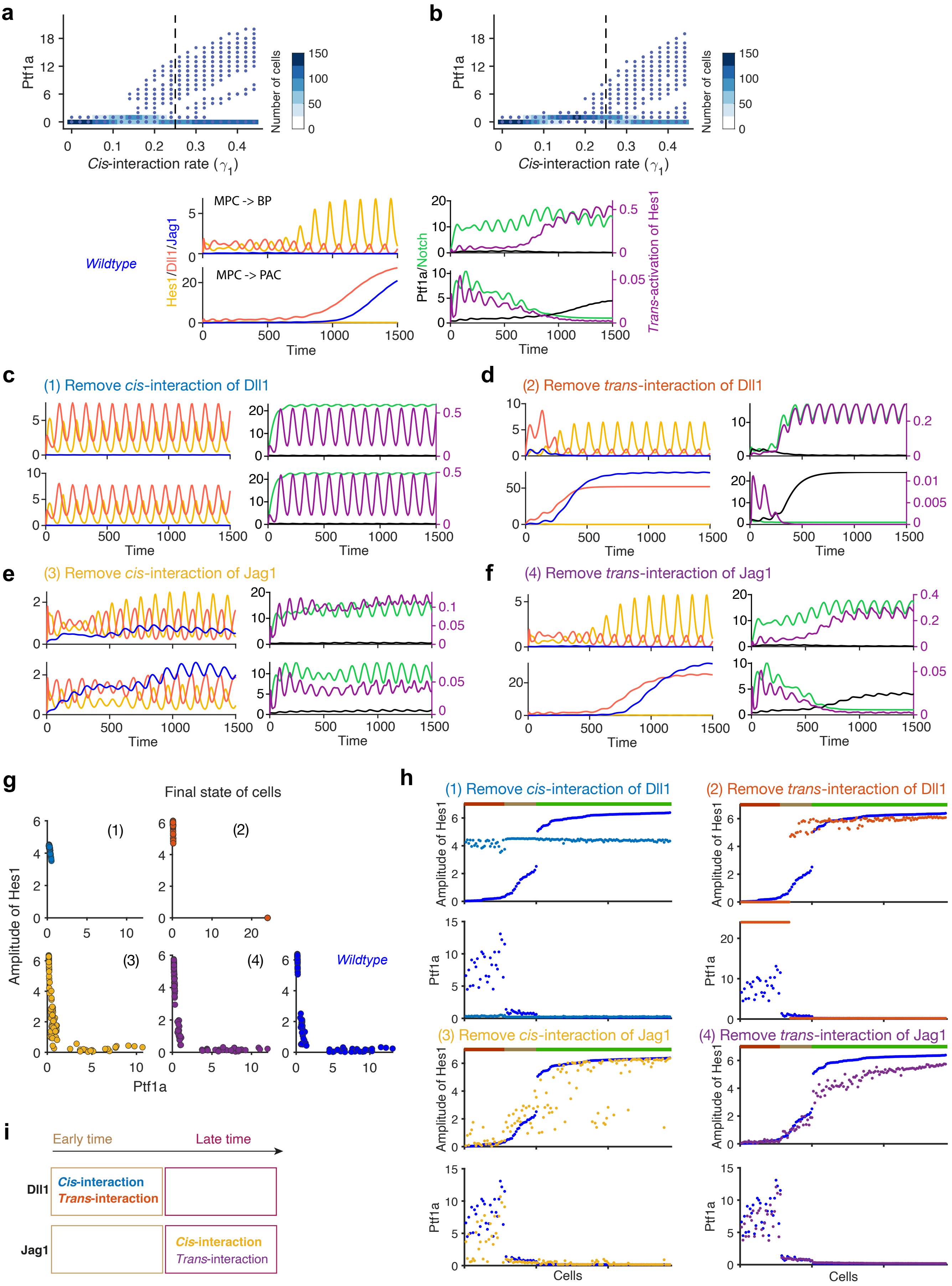
a
b
Ptf1a
Cis-interaction rate (γ_1)
Number of cells
Wildtype
MPC -> BP
MPC -> PAC
Hes1/Dll1/Jag1
Ptf1a/Notch
Trans-activation of Hes1
Time
c
(1) Remove cis-interaction of Dll1
d
(2) Remove trans-interaction of Dll1
e
(3) Remove cis-interaction of Jag1
f
(4) Remove trans-interaction of Jag1
g
Final state of cells
Amplitude of Hes1
Ptf1a
Wildtype
h
(1) Remove cis-interaction of Dll1
(2) Remove trans-interaction of Dll1
(3) Remove cis-interaction of Jag1
(4) Remove trans-interaction of Jag1
Cells
i
Early time
Late time
Dll1
Jag1
Cis-interaction
Trans-interaction
Cis-interaction
Trans-interaction

Supplementary Fig. 5. Jag1 bifurcates cell fates by mediating strong *Cis*-interaction

a Expression of Ptf1a in cells of simulation with different *cis*-interaction rates in E12.5 wildtype pancreatic cells. **b** Expression of Ptf1a in cells of simulation with different *cis*-interaction rates in *Jag1* deficient pancreas. **c–f** Examples of dynamics in the early time when removing *cis*-interaction of Dll1, *trans*-interaction of Dll1, *cis*-interaction of Jag1, or *trans*-interaction of Jag1 respectively. **g** Scatter plot of amplitude of Hes1 and expression of Ptf1a at final stage in cells: Dodger blue: without *cis*-interaction of Dll1, red: without *trans*-interaction of Dll1, yellow: without *cis*-interaction of Jag1, purple: without *trans*-interaction of Jag1. **h** Amplitude of Hes1 and Ptf1a at final state in each cell are compared between the four different conditions and *Wildtype* (blue) respectively. The colorbar at the top of each subfigure represents the cell fates of the cells in *Wildtype*. **i** Summary the roles of Dll1 and Jag1 at different time.

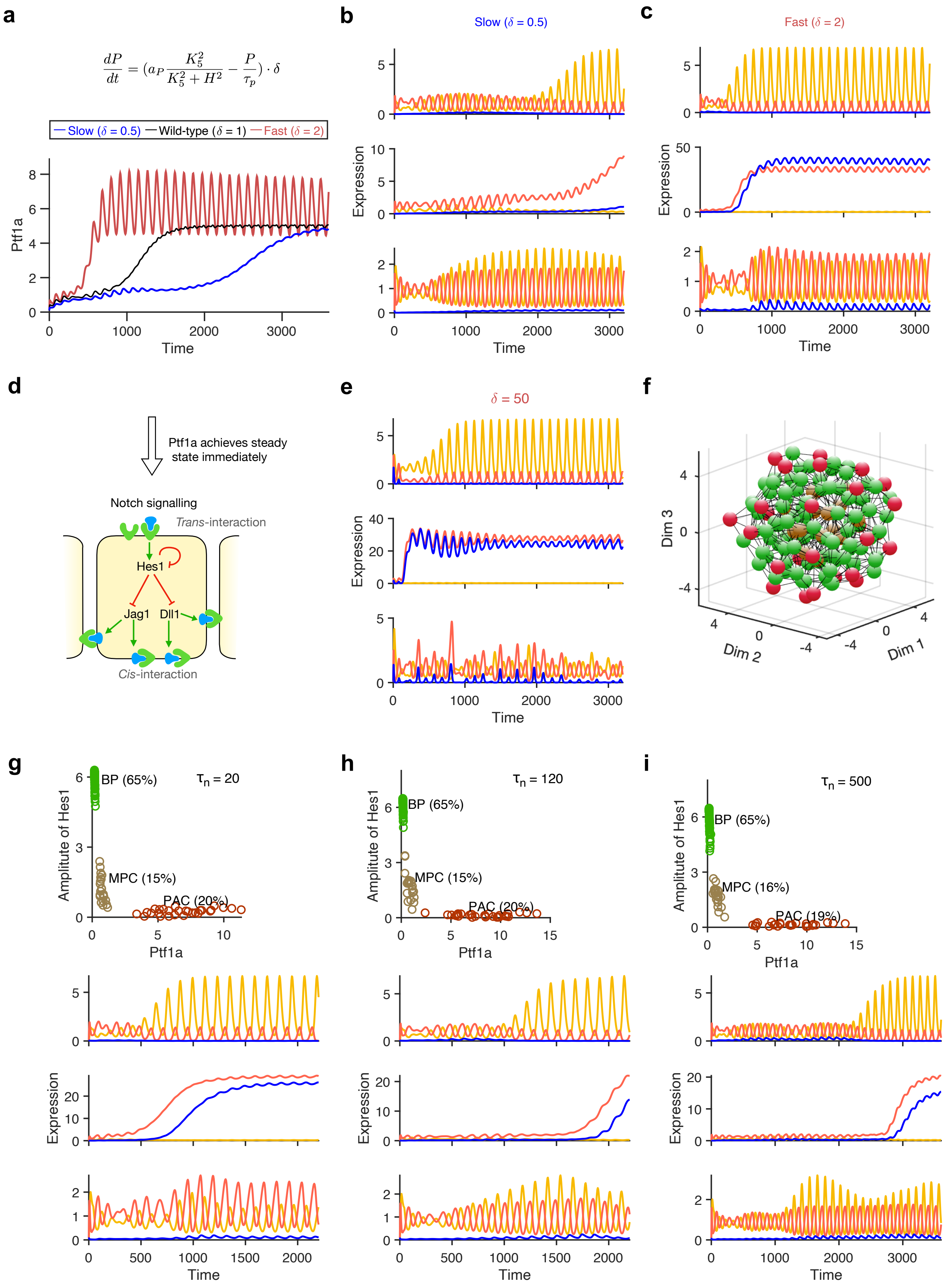
a
$$\frac{dP}{dt} = (a_P \frac{K_5^2}{K_5^2 + H^2} - \frac{P}{\tau_p}) \cdot \delta$$
Slow (δ = 0.5)
Wild-type (δ = 1)
Fast (δ = 2)
Ptf1a
Time
b
Slow (δ = 0.5)
Expression
Time
c
Fast (δ = 2)
Expression
Time
d
Ptf1a achieves steady state immediately
Notch signalling
Trans-interaction
Hes1
Jag1
Dll1
Cis-interaction
e
δ = 50
Expression
Time
f
Dim 3
Dim 2
Dim 1
g
BP (65%)
τn = 20
MPC (15%)
PAC (20%)
Amplitute of Hes1
Ptf1a
Expression
Time
h
BP (65%)
τn = 120
MPC (15%)
PAC (20%)
Amplitute of Hes1
Ptf1a
Expression
Time
i
BP (65%)
τn = 500
MPC (16%)
PAC (19%)
Amplitute of Hes1
Ptf1a
Expression
Time

Supplementary Fig. 6. Time scale of Ptf1a determines the timing of cell fate segregation. **a** Model modification changing the time scale of Ptf1a. The maximum production and degradation rates of Ptf1a are decreased or increased with a parameter, δ. $\delta < 1$ indicates the dynamic of Ptf1a become slower, and $\delta > 1$ indicates the dynamic of Ptf1a become faster. **b–c** Examples for dynamics of gene expression in cells with different time scale of Ptf1a. Yellow curves: Hes1, Red curves: Dll1, and blue curves: Jag1. **d** Equivalent model with very fast Ptf1a dynamics. **e** Examples for dynamics of gene expression in cells with very fast Ptf1a ($\delta = 50$). Jag1 also shows oscillatory dynamics in MPC cells. Yellow curves: Hes1, Red curves: Dll1, and blue curves: Jag1. **f** Cell fate distribution on 3D structure with very fast Ptf1a time scale. **g–i** Free notch receptor degradation time (τ_n) affects timing of cell fate segregation but not cell proportions.

a

$$\frac{dH}{dt} = a_H \frac{K_1^2}{K_1^2 + H(t-\tau_0)^2} \frac{\gamma_2^2([ND_t]+[NJ_t])^2}{\gamma_2^2([ND_t]+[NJ_t])^2 + K_2^2} - \frac{H}{\tau_h}$$

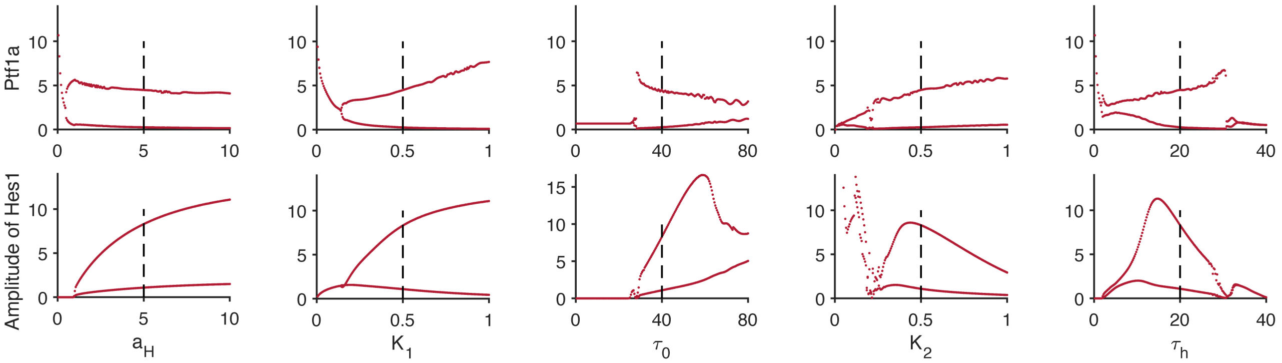

b

$$\frac{dD}{dt} = a_D \frac{K_3^2}{K_3^2 + H^2} + a_w \frac{P^2}{P^2 + K_6^2} - \gamma_1[ND] - \gamma_2[DN_t] - \frac{D}{\tau_d}$$

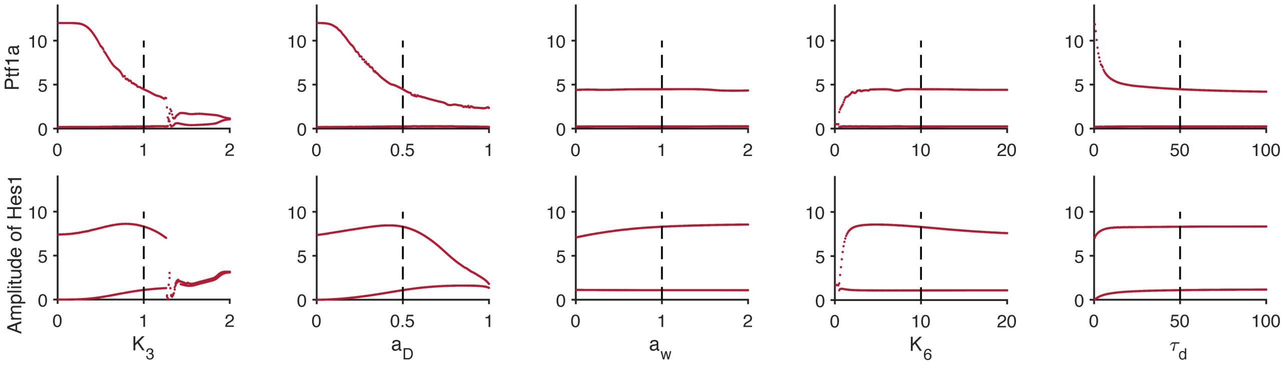

c

$$\frac{dJ}{dt} = a_J \frac{P^2}{P^2 + K_4^2} - \gamma_1[NJ] - \gamma_2[JN_t] - \frac{J}{\tau_j}$$

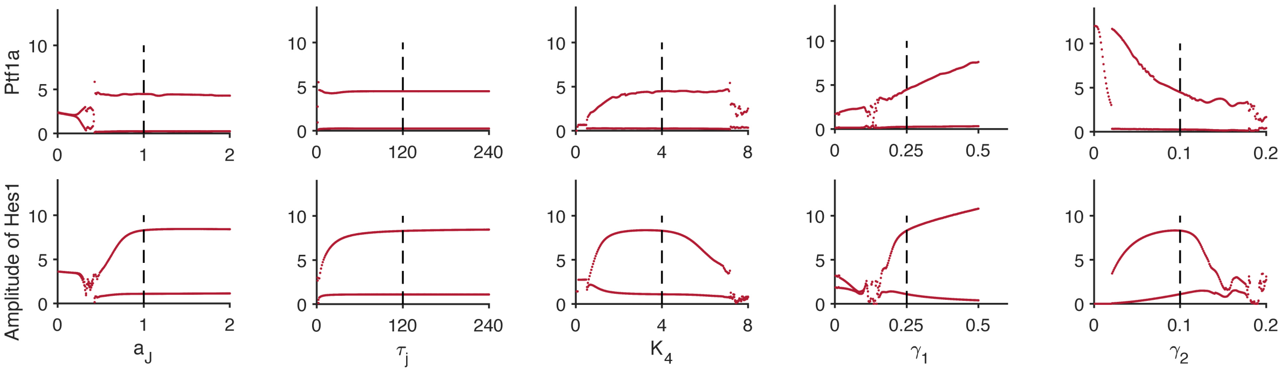

d

$$\frac{dN}{dt} = a_N - \gamma_1[ND] - \gamma_1[NJ] - \gamma_2[ND_t] - \gamma_2[NJ_t] - \frac{N}{\tau_n}$$

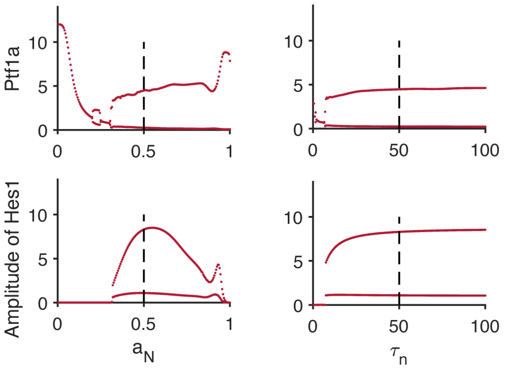

e

$$\frac{dP}{dt} = a_P \frac{K_5^2}{K_5^2 + H^2} - \frac{P}{\tau_p}$$

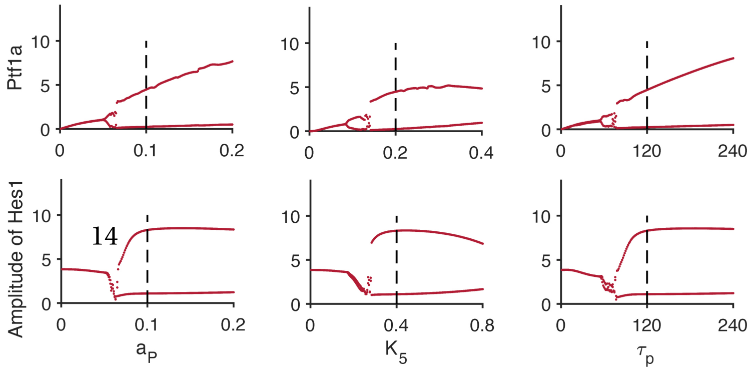

Supplementary Fig. 7. Parameter sensitivity analysis with the two-cell system. **a** Parameters relevant to Hes1. **b** Parameters relevant to Dll1. **c** Parameters relevant to Jag1. **d** Parameters relevant to Notch. **e** Parameters relevant to Ptf1a. Each parameter is changed in a range from 1% to 200% of it. The Ptf1a and the amplitude of Hes1 oscillation in the final state of the cells with each value of the parameters are presented.

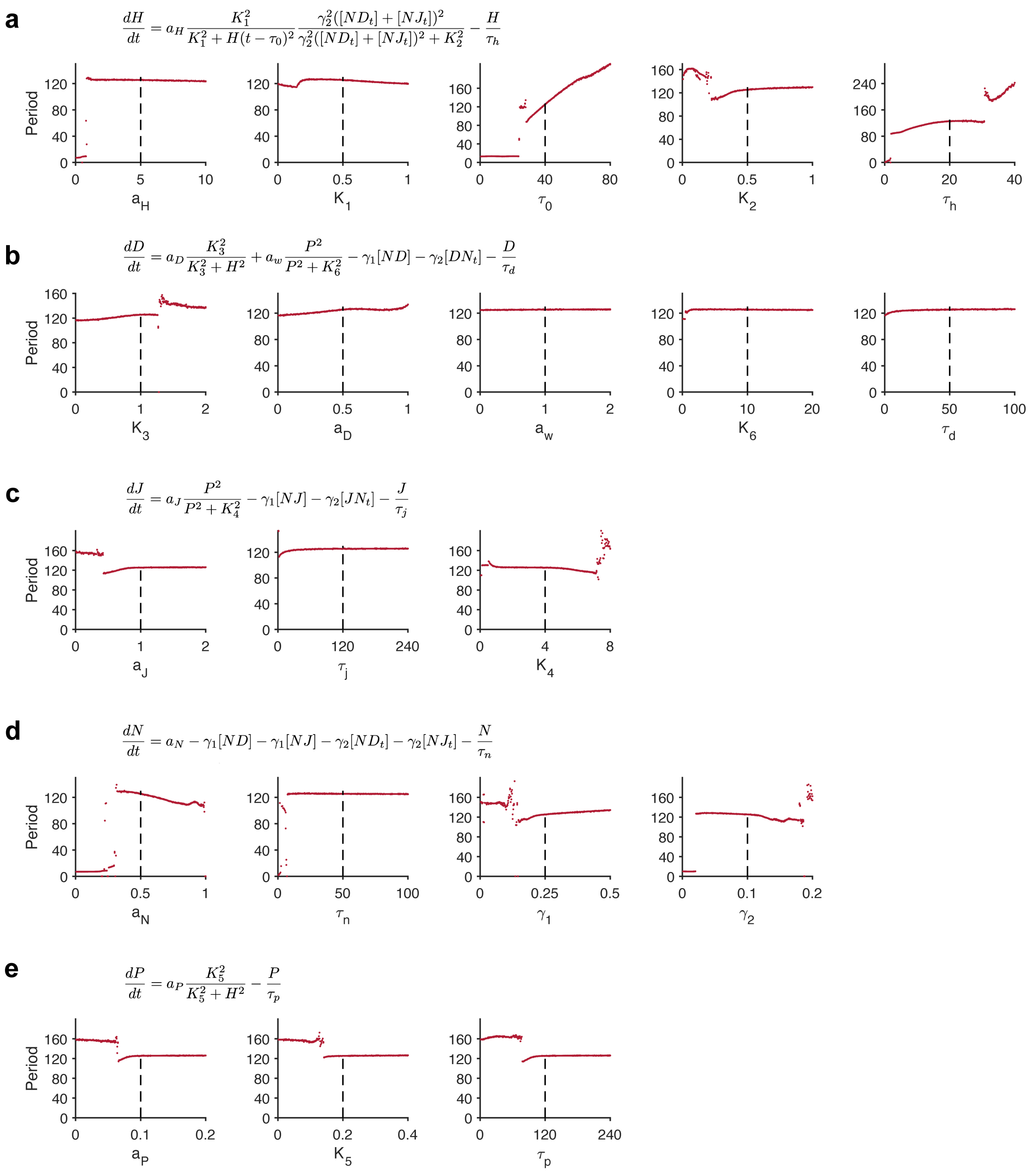

Supplementary Fig. 8. Parameter sensitivity analysis with the two-cell system regarding the period of Hes1 oscillation. **a** Parameters relevant to Hes1. **b** Parameters relevant to Dll1. **c** Parameters relevant to Jag1. **d** Parameters relevant to Notch. **e** Parameters relevant to Ptf1a. Each parameter is changed in a range from 1% to 200% of it. The period of Hes1 oscillation in the final state of the cells with each value of the parameters is presented.
