# Supplementary material for: Jag1-Notch cis-interaction determines cell fate segregation in pancreatic development
Source: Nat Commun. 2023 Jan 21;14:348. doi: 10.1038/s41467-023-35963-w (PMC9867774; doi:10.1038/s41467-023-35963-w)
Supplement: Supplementary file 3 — Description of Additional Supplementary Files [file 41467_2023_35963_MOESM3_ESM.pdf]

Description of Additional Supplementary Files

File Name: Supplementary Movie 1
Description: gene expression in wild type.

File Name: Supplementary Movie 2
Description: gene expression with DAPT treatment.

File Name: Supplementary Movie 3
Description: gene expression with MLN4924 treatment.

File Name: Supplementary Movie 4
Description: gene expression in *Dll1 deficient*.

File Name: Supplementary Movie 5
Description: gene expression in *Jag1 deficient*.

In all movies, the left panels show temporal expression of Hes1 (green) and Ptf1a (red) in each cell on 3D spatial structure, and the right panels show temporal expression of Dll1 and Jag1 in each cell with different colors.
