# Supplementary material for: Jag1-Notch cis-interaction determines cell fate segregation in pancreatic development
Source: Nat Commun. 2023 Jan 21;14:348. doi: 10.1038/s41467-023-35963-w (PMC9867774; doi:10.1038/s41467-023-35963-w)
Supplement: Supplementary file 9 — Reporting Summary [file 41467_2023_35963_MOESM9_ESM.pdf]

nature portfolio

Corresponding author(s): Mogens H. Jensen

Last updated by author(s): Dec 15, 2022

Reporting Summary

Nature Portfolio wishes to improve the reproducibility of the work that we publish. This form provides structure for consistency and transparency in reporting. For further information on Nature Portfolio policies, see our Editorial Policies and the Editorial Policy Checklist.

Statistics

For all statistical analyses, confirm that the following items are present in the figure legend, table legend, main text, or Methods section.

n/a	Confirmed	
☐	☒	The exact sample size (n) for each experimental group/condition, given as a discrete number and unit of measurement
☐	☒	A statement on whether measurements were taken from distinct samples or whether the same sample was measured repeatedly
☐	☒	The statistical test(s) used AND whether they are one- or two-sided<br>*Only common tests should be described solely by name; describe more complex techniques in the Methods section.*
☒	☐	A description of all covariates tested
☒	☐	A description of any assumptions or corrections, such as tests of normality and adjustment for multiple comparisons
☐	☒	A full description of the statistical parameters including central tendency (e.g. means) or other basic estimates (e.g. regression coefficient) AND variation (e.g. standard deviation) or associated estimates of uncertainty (e.g. confidence intervals)
☐	☒	For null hypothesis testing, the test statistic (e.g. F, t, r) with confidence intervals, effect sizes, degrees of freedom and P value noted<br>*Give P values as exact values whenever suitable.*
☒	☐	For Bayesian analysis, information on the choice of priors and Markov chain Monte Carlo settings
☒	☐	For hierarchical and complex designs, identification of the appropriate level for tests and full reporting of outcomes
☒	☐	Estimates of effect sizes (e.g. Cohen's d, Pearson's r), indicating how they were calculated

Our web collection on statistics for biologists contains articles on many of the points above.

Software and code

Policy information about availability of computer code

Data collection	Data from simulations in this manuscript are collected with MATLAB 2019a (64-bit). The code generated during and/or analysed during the current study is available at https://doi.org/10.5281/zenodo.7071389
Data analysis	Data from simulations in this manuscript are analysed with MATLAB 2019a (64-bit) and GraphPad Prism 9.4.1.

For manuscripts utilizing custom algorithms or software that are central to the research but not yet described in published literature, software must be made available to editors and reviewers. We strongly encourage code deposition in a community repository (e.g. GitHub). See the Nature Portfolio guidelines for submitting code & software for further information.

Data

Policy information about availability of data

All manuscripts must include a data availability statement. This statement should provide the following information, where applicable:

- Accession codes, unique identifiers, or web links for publicly available datasets
- A description of any restrictions on data availability
- For clinical datasets or third party data, please ensure that the statement adheres to our policy

All data generated or analysed during this study are included in this published article (and its supplementary information files).

Field-specific reporting

Please select the one below that is the best fit for your research. If you are not sure, read the appropriate sections before making your selection.

☒ Life sciences ☐ Behavioural & social sciences ☐ Ecological, evolutionary & environmental sciences

For a reference copy of the document with all sections, see nature.com/documents/nr-reporting-summary-flat.pdf

Life sciences study design

All studies must disclose on these points even when the disclosure is negative.

Sample size	No sample-size calculation was performed. Sample sizes were estimated on the basis of previous studies using similar methods and analyses that are widely published (e.g. Seymour et al. 2020, Dev Cell 52: 731-47; Katsumoto et al. 2022, Nat Commun 13:6255)
Data exclusions	No data were excluded from the analyses.
Replication	Experimental data were replicated using at least three independent biological samples (stated in the figure legends), except for a few IF marker analyses, where two biological replicates were assessed.
Randomization	All wild-type samples were allocated into experimental groups randomly. Mutant embryos were allocated into experimental groups based on genotyping on genomic DNA.
Blinding	No blinding was used in these experiments because the same investigator designed and conducted the experiments including the appropriate controls.

Reporting for specific materials, systems and methods

We require information from authors about some types of materials, experimental systems and methods used in many studies. Here, indicate whether each material, system or method listed is relevant to your study. If you are not sure if a list item applies to your research, read the appropriate section before selecting a response.

Materials & experimental systems

n/a	Involved in the study	
☐	☒	Antibodies
☒	☐	Eukaryotic cell lines
☒	☐	Palaeontology and archaeology
☐	☒	Animals and other organisms
☒	☐	Human research participants
☒	☐	Clinical data
☒	☐	Dual use research of concern

Methods

n/a	Involved in the study	
☒	☐	ChIP-seq
☒	☐	Flow cytometry
☒	☐	MRI-based neuroimaging

Antibodies

Antibodies used

Goat polyclonal anti-Pdx1 (IF 1:10000, WB 1:5000); Beta Cell Biology Consortium (BCBC); Cat#AB2027.
Guinea pig polyclonal anti-Sox9 (IF 1:2000); BCBC (Kind gift from Dr. Ole Madsen); N/A.
Rabbit polyclonal anti-Sox9 (IF 1:1000); Merck Millipore; Cat#AB5535.
Rabbit polyclonal anti-Ptf1a (IF 1:3000); BCBC; Cat#AB2153.
Guinea pig polyclonal anti-Ptf1a (IF 1:5000); Kind gift from Dr. Jane E. Johnson; Hori et al. 2008, Genes Dev 22: 166-78.
Mouse monoclonal anti-Nkx6-1, clone # F55A10 (IF 1:500); DSHB; Cat#F55A10 .
Rabbit polyclonal anti-Nkx6-1 (IF 1:2000); BCBC; Cat#AB1069.
Rabbit polyclonal anti-Ngn3 (IF 1:4000 for sections, 1:10000 for whole mount); BCBC; Cat#AB2011.
Guinea pig polyclonal anti-Glucagon (IF 1:4000 for sections, 1:10000 for whole mount); Millipore (Merck); Cat#4031-01F.
Rabbit polyclonal anti-Glucagon (IF 1:2000); Cell Signaling Technology; Cat#2760.
Rat monoclonal anti-E-Cadherin (Cdh1), clone # ECCD-2 (IF 1:1000); ThermoFisher; Cat#13-1900.
Sheep polyclonal anti-Dll1 (IF 1:200); R&D Systems; Cat#AF3970.
Goat polyclonal anti-Jag1 (IF 1:200, WB 1:500); Santa Cruz Biotechnology; Cat#sc-6011.
Rabbit monoclonal anti-Jag1, clone # 28H8 (IF 1:50); Cell Signaling Technology; Cat#2620.
Rabbit monoclonal anti-Hes1, clone # D6P2U (IF 1:200); Cell Signaling Technology; Cat#11988.
Mouse monoclonal anti-pan-Actin (WB 1:4000); ThermoFisher; Cat# MA5-11869.

Validation

Jag1, Dll1 and Hes1 antibodies are validated in Seymour et al. (2020) Dev Cell 52: 731-47. All other antibodies are used extensively in the field and fully validated by numerous studies (e.g. Jørgensen et al. 2018, Development 145: dev163568; Bechard et al. 2016, Genes Dev 30: 1852-65; Shih et al. 2012, Development 139: 2488-99; Schaffer et al. 2010, Dev Cell 18: 1022-29).

Animals and other organisms

Policy information about studies involving animals; ARRIVE guidelines recommended for reporting animal research

Laboratory animals	Mus musculus, mixed 129Sv-C57Bl6/J background. Embryos were collected at E10.5, E12.5 and E13.5 and their sex was not determined.
Wild animals	The study did not involve wild animals.
Field-collected samples	The study did not involve field-collected samples.
Ethics oversight	All mice were housed at the Department for Experimental Medicine at the University of Copenhagen as approved by ''Miljø- og fødevareministeriet - Dyreforsøgstilsynet'' who also approved the experiments involving animals.

Note that full information on the approval of the study protocol must also be provided in the manuscript.
