# Supplementary figures and images for: Jag1-Notch cis-interaction determines cell fate segregation in pancreatic development
Source: Nat Commun. 2023 Jan 21;14:348. doi: 10.1038/s41467-023-35963-w (PMC9867774; doi:10.1038/s41467-023-35963-w)

anti-Ptf1a

anti-Jag1

anti-Actin

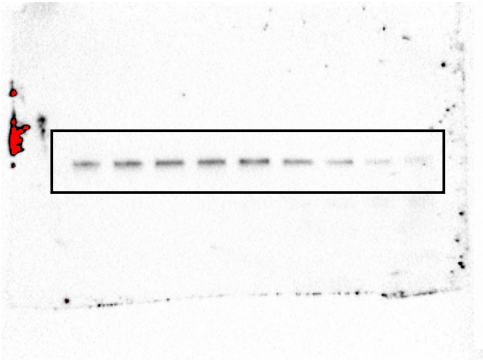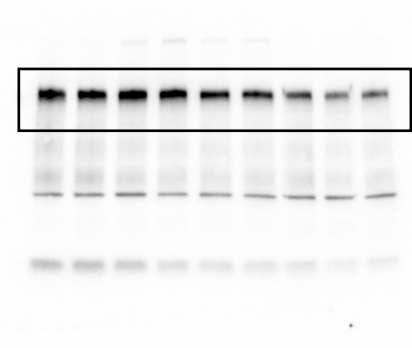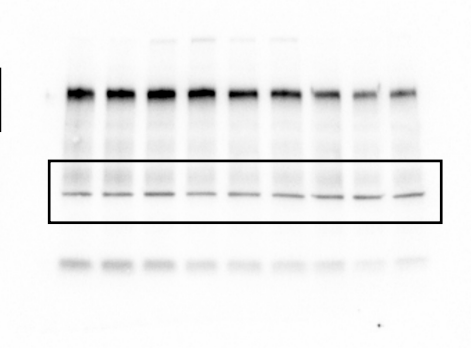

Exp. 1

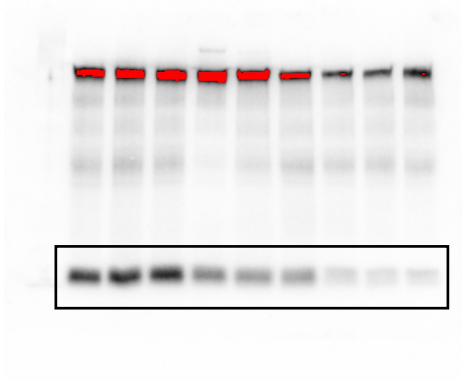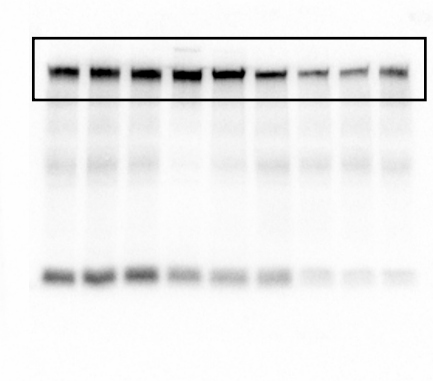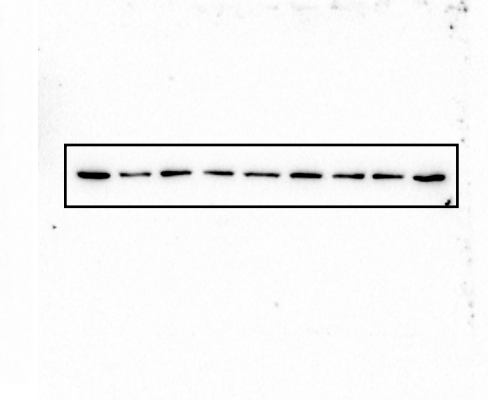

Exp. 2

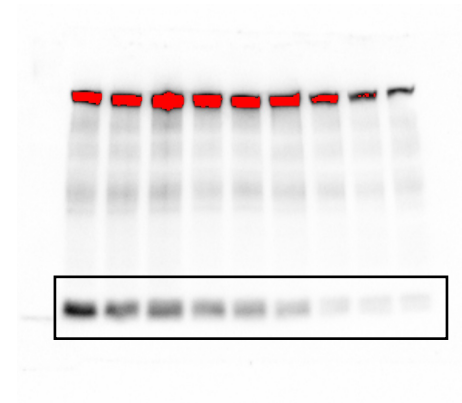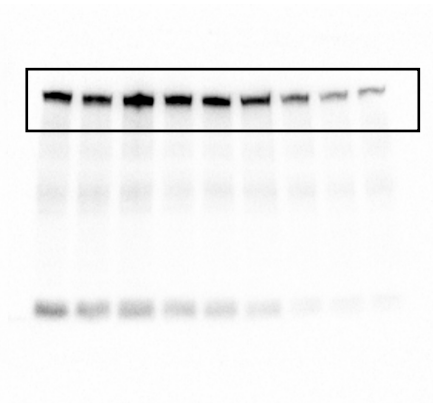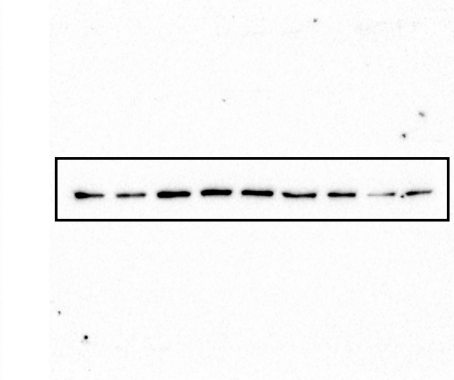

Exp. 3

Supplement: Supplementary file 10 — Source Data [file 41467_2023_35963_MOESM10_ESM.zip › Source_images_Fig_S1b.pdf]
